# Sleep, Sedentary Time and Physical Activity Levels in Children with Cystic Fibrosis

**DOI:** 10.3390/ijerph19127133

**Published:** 2022-06-10

**Authors:** Mayara S. Bianchim, Melitta A. McNarry, Alan R. Barker, Craig A. Williams, Sarah Denford, Anne E. Holland, Narelle S. Cox, Julianna Dreger, Rachel Evans, Lena Thia, Kelly A. Mackintosh

**Affiliations:** 1 Applied Sports, Technology, Exercise and Medicine Research Centre, Bay Campus, Swansea, Wales SA1 8EN, UK; tuca_may@hotmail.com (M.S.B.); k.mackintosh@swansea.ac.uk (K.A.M.); 2 Children’s Health and Exercise Research Centre, University of Exeter, Exeter EX1 2LU, UK; a.r.barker@exeter.ac.uk (A.R.B.); c.a.williams@exeter.ac.uk (C.A.W.); sarah.denford@bristol.ac.uk (S.D.); 3 Respiratory Research@Alfred, Department of Immunology, Monash University, Melbourne, VIC 3004, Australia; anne.holland@monash.edu (A.E.H.); narelle.cox@monash.edu (N.S.C.); jdreger@ucalgary.ca (J.D.); 4 Institute for Breathing and Sleep, Melbourne, VIC 3004, Australia; 5 Physiotherapy, Alfred Health, Melbourne, VIC 3004, Australia; 6 Paediatric Department, Morriston Hospital, Swansea SA6 6NL, UK; rachel.evans8@wales.nhs.uk; 7 Department of Paediatric Respiratory Medicine and Cystic Fibrosis Unit, Noah’s Ark Children’s Hospital for Wales, Cardiff CF14 4XW, UK; lena.thia@wales.hns.uk

**Keywords:** threshold, MVPA, accelerometry, lung function, clinical, ENMO

## Abstract

The aim of this study was to compare the use of generic and cystic fibrosis (CF)-specific cut-points to assess movement behaviours in children and adolescents with CF. Physical activity (PA) was assessed for seven consecutive days using a non-dominant wrist-worn ActiGraph GT9X in 71 children and adolescents (36 girls; 13.5 ± 2.9 years) with mild CF. CF-specific and generic Euclidean norm minus one (ENMO) cut-points were used to determine sedentary time (SED), sleep, light physical activity (LPA), moderate physical activity and vigorous physical activity. The effect of using a CF-specific or generic cut-point on the relationship between PA intensities and lung function was determined. Movement behaviours differed significantly according to the cut-point used, with the CF-specific cut-points resulting in less time asleep (−31.4 min; *p* < 0.01) and in LPA (−195.1 min; *p* < 0.001), and more SED and moderate-to-vigorous PA (159.3 and 67.1 min, respectively; both *p* < 0.0001) than the generic thresholds. Lung function was significantly associated with LPA according to the CF-specific cut-points (r = 0.52; *p* = 0.04). Thresholds developed for healthy populations misclassified PA levels, sleep and SED in children and adolescents with CF. This discrepancy affected the relationship between lung function and PA, which was only apparent when using the CF-specific cut-points. Promoting LPA seems a promising strategy to enhance lung function in children and adolescents with CF.

## 1. Introduction

Cystic fibrosis (CF) is the most common autosomal inherited condition in the Caucasian population, affecting 70,000 people worldwide [1]. Respiratory dysfunction manifests in early childhood and recurrent infections lead to the development of bronchiectasis, culminating in progressive lung function impairment [2]. Progressive lung impairment is one of the most important features of this systemic condition, which often culminates in respiratory failure. Whilst exercise intolerance in CF is multifactorial, involving chronic inflammation, poor nutritional status and muscle weakness, the main factor is the progressive airway disease with subsequent abnormal ventilatory response to exercise [3,4]. Limited exercise tolerance often leads to physical inactivity and the adoption of a sedentary lifestyle, which is associated with negative health implications [5]. In contrast, physical activity (PA), particularly moderate-to-vigorous physical activity (MVPA), is considered a key element in CF care, and is associated with multiple benefits [6]. Specifically, regular practice of PA is associated with a slower decline in lung function and enhanced nutritional status and bone mineral density, and better quality of life [7,8,9,10,11,12].

There is a dearth of research assessing PA in children and adolescents with CF using accelerometers, despite their superior validity and reliability relative to self-report methods [13]. Specifically, in addition to contradictory findings regarding the total volume of PA in those with CF compared to their healthy peers, there is also no consensus regarding the intensity distribution, with some studies reporting that children and adolescents with CF accumulated less vigorous physical activity (VPA) than their healthy peers [14,15,16], whereas Selvadurai et al. [17] and Mackintosh, Ridgers, Evans and McNarry [18] reported no difference in VPA. These equivocal findings may be related to inter-study differences in the study population and protocols. For example, age [19,20] and sex [17] affect the PA levels of children and adolescents with CF but the majority of previous studies have not accounted for these factors [14,21].

The lack of consensus regarding PA levels in those with CF may also be due to methodological limitations associated with earlier studies, such as the use of generic cut-points [18]. Specifically, the use of cut-points developed for healthy populations may be associated with the misclassification of PA intensities when applied to clinical populations, such as CF [18,22]. Indeed, children with CF expend more energy for a given activity, including rest, than their healthy peers due to impaired metabolic and ventilatory responses [23]. Research to date has therefore potentially over-estimated time spent in light physical activity (LPA) and under-estimated time spent in MVPA in people with CF, which has potentially led to erroneous conclusions regarding the relationship of PA with health [14,21]. Such misclassifications may explain the higher LPA and lower MVPA reported in children with CF relative to their healthy counterparts [14,15]. Additionally, previous studies have relied on count-based cut-points, for which limitations are widely recognised. Specifically, Schmiedek, et al. [24] highlighted that vital information for classifying PA may be lost during the data reduction process involved in converting raw accelerometer data into counts. For example, information regarding subtle movements that may enable the differentiation between standing and non-wear time can be lost during the reduction to counts [24]. In accord, the use of cut-points developed from raw acceleration metrics, such as Euclidean norm minus one (ENMO), provides superior accuracy in comparison to counts [24]. Therefore, the use of CF-specific raw acceleration cut-points has the potential to advance our current knowledge of the PA levels of children and adolescents with CF, and, importantly, the relationship of these PA levels with associated health outcomes.

The relationship between PA levels and health in those with CF largely remains to be elucidated. Although higher PA levels are generally accepted to be associated with a slower decline in lung function in children with CF [7], the evidence regarding the optimal PA intensity remains equivocal. Specifically, Mackintosh, Ridgers, Evans and McNarry [18] reported that ‘high’ LPA was the only predictor of lung function but, in contrast, others reported that VPA was primarily associated with lung function in children with CF [14,21]. The aim of this study was to identify whether PA classification discrepancies are attributable to the misclassification of PA and/or to a failure to account for key factors such as age and sex. The secondary aim of this study was to determine the influence of cut-point selection on the relationship between PA and lung function in those with CF.

## 2. Materials and Methods

### 2.1. Participants

Participants were recruited from paediatric CF services in Australia (*n* = 58) and South Wales (*n* = 35). Participants from Australia constituted the baseline of a randomised controlled trial intervention, detailed elsewhere [25]. Participants from Wales also constituted the baseline of a cross-sectional study published elsewhere [26]. Participants aged 7–18 years diagnosed as having CF through a new-born screening test, and/or those presenting CF-typical symptoms and either two pathological sweat tests or the identification of two CF-relevant mutations, were included. Exclusion criteria were the presence of multi-resistant bacteria, or being on the transplant list. Written informed assent and consent were obtained from all the participants and their parents/guardians, respectively. Ethics approval was obtained from the West of Scotland National Health Service Research Ethics Committee (18/WS/0032) and from the Human Research Ethics Committee at Alfred Health in Australia (HREC/16/Alfred/188; Project 7/17).

### 2.2. Measurements

Body mass and stature were measured to the nearest 0.1 kg and 0.1 cm, respectively, and body mass index (BMI) was calculated, with BMI z-scores determined using the World Health Organization reference data [27]. Lung function was assessed through standard spirometry (Metamax 3B, Cortex Biophysik GmbH, Germany) using a forced vital capacity manoeuvre in accordance with the American Thoracic Society and European Respiratory Society standards [28,29]. The forced expiratory volume in the first second (FEV_1_) was determined and percentage of predicted values (FEV_1_%_predicted_) estimated using age-, sex- and height-specific equations [30], which were subsequently used to classify disease severity as mild (FEV_1_ ≥ 70%_predicted_), moderate (FEV_1_ 40–69%_predicted_), or severe ≤ 40%; [31].

#### Accelerometry

Physical activity was measured using the ActiGraph GT9X Link (ActiGraph, Pensacola, FL, USA) worn on the non-dominant wrist for seven consecutive days. Participants were instructed to wear the monitors at all times, including during sleep. Accelerometer data were downloaded as 100 Hz. gt3x files using ActiLife V 6.10.2 software, and subsequently converted to time-stamp free. csv files for processing using the GGIR package V 1.2–0; [32] in R statistical software (R V3.1.2 Foundation for Statistical Computing, Vienna, Austria). The GGIR package was used to auto-calibrate the data, detect abnormal values and non-wear time, and extract the ENMO. Subsequently, the ENMO values, expressed as mg, were further reduced to 5-s epochs [33].

All files with a post-calibration error greater than 0.02 g or less than three valid days, including one weekend day, were excluded from subsequent analyses [34]. At least 16-h of wear-time per day was required to be considered valid [32]. The non-wear detection is described in detail elsewhere [32]. ENMO was calculated for time accumulated sedentary and in MPA and VPA using the recently developed CF-specific cut-points (38.4 mg, 60.2 mg and 115.3 mg, respectively) [35] and a generic cut-point developed from healthy children (35.6 mg; 201.4 mg, 707.0 mg, respectively) [36,37].

The integrated algorithm for sleep analysis developed by van Hees et al., and incorporated into GGIR was used to estimate sleep. Essentially, sleep time was estimated in minutes per day as any period of sustained inactivity with no change of more than five degrees in the monitor angle during a nocturnal sleep window, identified using the van Hees, et al. [38] heuristic algorithm for wrist-worn accelerometers. The sleep/wake patterns were visually inspected to confirm that the nocturnal sleep pattern was correctly estimated; it is usually observed in periods of lower frequency of wrist movement [39].

### 2.3. Statistical Analysis

Descriptive statistics (mean ± standard deviation (SD)). The effect of cut-point selection, age, sex, FEV_1_%_predicted_ and their interaction on the time spent in each PA intensity and sleep were assessed using linear mixed methods, with wear-time as a random effect at the participant level. A stepwise linear regression explored the association between FEV_1_%_predicted_ and time spent in different PA intensities, adjusting for key confounding factors (age, sex, BMI, genotype, wear-time). Finally, a chi-square test was conducted to compare the impact of the cut-points in determining whether participants met PA guidelines defined as an average of 60 min/day of MVPA [40]. All analyses were performed using SPSS version 23.0 (IBM Corp., Armonk, NY, USA). Statistical significance was accepted when *p* ≤ 0.05.

## 3. Results

A total of 93 participants with CF (36 girls; 13.5 ± 2.9 years) participated in the study, of which 42% were homozygous for ΔF508 mutation and 20% had cystic fibrosis-related diabetes. Forty-six participants were classed as having mild CF, 24 as having moderate and one as having severe CF. In total, 71 participants (Table 1) were included in the final analysis after excluding (*n* = 22) those that did not meet the wear-time criteria. No significant differences were found in demographic, anthropometric or lung function characteristics for those included or excluded from the analysis.

Boys accumulated more MPA and MVPA overall and on weekdays in comparison to girls. Girls accumulated more MVPA on weekends and had significantly higher LPA, overall and on weekdays for CF-specific cut-points [38] in comparison to boys. Additionally, girls accumulated less sleep and more SED during the week for Hildebrand et al. [36,37] cut-points (Table 2) in comparison to boys. In total, 33 participants (46.5%) met the PA guidelines [40], when using Hildebrand et al., cut-points, whereas 64 (90%) participants met the recommendations when using CF-specific cut-points.

The time spent in all PA intensities and asleep significantly differed according to cut-point selection, independent of age, sex or FEV_1_%_predicted_. Specifically, SED, MPA, VPA and MVPA were significantly higher, and sleep and LPA were lower for CF-specific cut-points. The PA levels according to sex and cut-points are presented in Table 2. All physical activity intensities and sleep significantly differed according to cut-points in the linear mixed model analysis (Table 3), independently of sex, age and FEV_1_%_predicted_. Specifically, time spent on sleep and LPA was significantly greater when using Hildebrand et al., cut-points. In contrast, Hildebrand et al., cut-points yielded lower time spent in SED, MPA, VPA and MVPA.

Age emerged as an important predictor of FEV_1_, independent of the cut-points utilised. Specifically, age was associated with sleep, MPA, VPA and MVPA (β = −7.7, *p* = 0.03; β = −6.9, *p* = 0.0001; β = −4.1, *p* = 0.003; β = −11.1, *p* = 0.0001, respectively). When using CF-specific cut-points, only LPA was associated with FEV_1_ (r = 0.52, β = −0.25, *p* = 0.04) after adjusting for key confounders. In the unadjusted model, SED was associated with FEV_1_ (r = 0.41, β = 0.41, *p* = 0.03), but this relationship was ameliorated when age, BMI, wear-time, genotype and sex were accounted for. No significant association was found between FEV_1_ and PA when using Hildebrand et al., cut-points. Finally, an association between FEV_1_ and sleep (r = 0.29, β = −0.29, *p* = 0.038) was found for both thresholds, but it was not sustained after adjusting the model.

## 4. Discussion

This study sought to compare the use of generic raw accelerometry cut-points and CF-specific cut-points on the PA levels of children and adolescents with CF. The CF-specific cut-points resulted in significantly more time spent in MPA, VPA, MVPA and sedentary and less time asleep and in LPA. The relationship between lung function and PA was only apparent with the condition-specific thresholds, with FEV_1_ dependent on LPA.

The significant discrepancies observed between cut-points have important implications regarding the interpretation of previous research that utilised generic thresholds to estimate PA levels in youth with CF. Specifically, the use of a generic cut-point appears to underestimate SED and MPA and VPA levels, whilst over-estimating LPA. These findings are in agreement with the hypothesis proposed by Mackintosh, Ridgers, Evans and McNarry [18], suggesting that divergences in PA levels previously reported in CF resulted, at least in part, from the inappropriate use of generic cut-points and subsequent misclassification of MVPA as LPA. It is noteworthy that such misclassification may have affected previous comparisons between the relative PA levels of those with CF and healthy populations. Indeed, whilst some evidence showed that children with CF did not accumulate as much MVPA as their healthy peers [14,15,21], others found no differences [18]. The misclassification of MVPA also impacts conclusions regarding whether children and adolescents are meeting the recommended PA guidelines. Indeed, the present study demonstrated that only 46.5% of children met the guidelines when using the generic thresholds, in comparison to 90% when using the CF-specific cut-points. These findings raise important questions regarding the applicability of generic cut-points and subsequent PA recommendations to those with CF, suggesting a need for CF-specific recommendations regarding the optimal combination of PA intensities, frequency and duration.

Whilst the appropriate selection of cut-points is a major factor for accurately assessing PA in CF, this study also highlighted the importance of sex and type of day. Selvadurai et al. [17] similarly found that PA levels in CF are affected by sex and maturation, with girls decreasing their PA levels after the onset of puberty. In accord, the present study showed that age is a key predictor across all PA intensities, and it should be considered when assessing PA levels in CF, with younger children accumulating more MVPA. It is important to acknowledge, however, that chronological and biological age are not equivalent, and consequently, individuals with the same chronological age can significantly differ regarding biological maturity [41]. Therefore, further work is warranted to estimate the impact of biological age on PA levels in youth with CF. Furthermore, in accord with the current findings, previous studies have reported differences in how children and adolescents with CF accumulate PA levels during week and weekend days [15,18]. However, discordant with the present study, Mackintosh, Ridgers, Evans and McNarry [18] reported that children spent more time being sedentary and less time in MPA and LPA on weekend days. In contrast, but in agreement with the present study, Aznar et al. [15] found that children and adolescents with CF accumulated more SED and MVPA during weekdays than weekend days. This discrepancy could be attributed to cut-point misclassification or a failure to account for sex in earlier studies. More specifically, the present study showed that the amount of time accrued during week and weekend days varied according to sex and cut-points, with boys accumulating more MPA and MVPA during the week with CF-specific cut-points [35], and girls accumulating less sleep and more SED during the week for Hildebrand et al. [36,37] cut-points. In addition to highlighting the importance of the population-specific cut-points, these findings also indicate that PA interventions should be stratified by sex and type of day, given that boys and girls had significantly different PA across week and weekend days.

This study confirmed that the use of CF-specific cut-points produced significantly different PA levels in comparison with the generic thresholds. It is therefore postulated that previously reported associations between PA and lung function may subsequently be inaccurate. Whilst some evidence suggested, to a certain extent, a relationship between MVPA and FEV_1_ in children with CF [14,21], such findings were not corroborated by others [17]. It is noteworthy that the majority of previous research has not investigated the full spectrum of PA intensities, focusing solely on the relationship between MVPA and health [14,42]. Additionally, the use of self-reported measures, as commonly utilised in studies investigating PA levels in those with CF, is also likely to affect the association with health outcomes given their subjective nature and lack of accuracy. The limited research that has investigated the relationship between health and PA across the intensity spectrum similarly reported that LPA was significantly associated with FEV_1_ in children with CF [18], although it is important to acknowledge that these previous findings were based on count-based cut-points developed in healthy populations. Whilst the present study findings endorse that LPA was the most influential behaviour in terms of lung function, there is a growing body of evidence showing that both volume and intensity of weekly PA are important for health [43]. Therefore, despite the abundant evidence associating LPA with health [44,45], further work investigating the optimal weekly volume, frequency and duration that is associated with such benefits in CF is warranted.

The finding that CF-specific thresholds [35] yielded significantly higher levels of SED in comparison with Hildebrand et al. [36,37] cut-points warrants attention given the important role of this behaviour as an independent risk factor of all-cause mortality [46]. Specifically, this finding reflects the higher energetic demands for a given sedentary task that is associated with the pathophysiological alterations in CF, and it raises relevant questions regarding the definition of sedentary behaviour in this population. In particular, since the definition of sedentary behaviour is centred on an energetic cost threshold, i.e., <1.5 MET; [47], it could be hypothesised that this threshold should be higher in those with CF to account for the physiological limitations of this condition. Whilst it is beyond the scope of the present study to explore this contention, future research focused on SED in people with CF is warranted. Indeed, there is a worrying lack of research investigating the relationship between SED and disease severity in those with CF, with the limited evidence available demonstrating contradictory findings [15,18].

Strengths of the present study included it being the first study to assess PA using raw acceleration, CF-specific, cut-points. Furthermore, given that previous research indicated that PA is likely to differ across the week, the present study investigated total PA and SED across the week, as well as on week- and weekend days [15]. Despite that, this study is not without its limitations. It is pertinent to note that the children and adolescents included in this study were categorised as having mild CF and are unlikely to represent those with a more severe form of the condition. Moreover, this study adopted a cross-sectional design, and therefore, no causal inferences between PA and health can be established. We acknowledge that the lack of a non-CF control group may have limited further interpretations of the findings.

## 5. Conclusions

In conclusion, the present study revealed that previous research is likely to have misclassified PA levels in children and adolescents with CF, resulting in an underestimation of the percentage of those with CF who meet the current PA guidelines. This misrepresentation of PA levels in children with CF could have affected condition-specific PA recommendations and the design of interventions for this population. Future interventions should seek to promote LPA to increase lung function through a stratified approach according to sex.

## Figures and Tables

**Table 1:** Participant Characteristics by Sex.

Characteristics	Total (*n* = 71)	Girls (*n* = 36)	Boys (*n* = 35)
Age (years)	13.5 ± 2.9	13.5 ± 2.9	13.5 ± 2.8
Height (cm)	154.1 ± 14.9	151.7 ± 14.0	156.5 ± 15.7 *
Body mass (kg)	46.2 ± 14.6	44.9 ± 12.9	47.5 ± 16.2
BMI (kg·m^−2^)	19.0 ± 3.9	19.1 ± 2.9	18.9 ± 4.7
zBMI	−0.2 ± 1.0	−0.04 ± 0.8	−0.4 ± 1.14
FEV_1_ (L)	2.3 ± 0.8	2.1 ± 0.7	2.5 ± 0.8 *
FEV_1_%_predicted_	84 ± 21	83 ± 25	86 ± 18

Data are presented as mean ± SD. FEV_1_: forced expiratory volume in one second, FEV_1_%_predicted_: forced expiratory volume in one second predicted, BMI: body mass index, zBMI: z-scores body mass index. * Significant sex difference (*p* ≤ 0.05).

**Table 2:** Physical activity levels across weekdays, weekend days and overall (weekdays and weekend) by sex and cut-point.

	Generic *Hildebrand* et al.			CF-Specific *Bianchim* et al.		
Overall	Overall	Boys	Girls	Overall	Boys	Girls
**Sleep**	529.8 ± 86.9	525.8 ± 102.2	533.5 ± 69.5	501.6 ± 94.1	590.2 ± 117.05	507.4 ± 60.2
**SED**	384.8 ± 213.1	395.5 ± 223.8	375.0 ± 202.5	555.3 ± 150.8 #	576.4 ± 176.5 #	533.2 ± 129.3 #
**LPA**	414.8 ± 259.2	407.0 ± 273.4	422.1 ± 245.6	206.4 ± 73.5 #	188.1 ± 76.7 #+	226.2 ± 65.3 #
**MPA**	74.6 ± 59.6	75.8 ± 58.7	73.5 ± 60.4	126.3 ± 47.6 #	121.4 ± 50.3 #	131.7 ± 47.3 #
**VPA**	31.6 ± 37.9	35.7 ± 43.5	27.8 ± 31.4	50.2 ± 31.6 #	59.0 ± 36.2 #+	41.2 ± 25.4 #
**MVPA**	105.9 ± 91.5	111.5 ± 94.9	100.6 ± 88.4	176.5 ± 66.3 #	180.4 ± 73.1 #	172.9 ± 63.1 #
**Week days**						
**Sleep**	521.3 ± 153.2	556.9 ± 195.7	486.3 ± 93.1 +*	554.8 ± 129.4	594.5 ± 157.7+	514.9 ± 88.5
**SED**	451.6 ± 148.7	428.9 ± 136.5	474.1 ± 163.0 +*	543.6 ± 144.6 #	532.5 ± 161.4 #	554.4 ± 136.6 #
**LPA**	387.7 ± 221.1	373.6 ± 240.7	400.5 ± 206.9	215.5 ± 82.9 #	194.5 ± 87.0 #+	236.7 ± 78.7 #
**MPA**	77.5 ± 59.4	78.4 ± 57.1	77.7 ± 63.6	127.3 ± 49.6 #	124.2 ± 50.2 #*	130.9 ± 51.7 #
**VPA**	30.1 ± 36.7	35.6 ± 41.3	25.0 ± 29.6	49.1 ± 31.6 #	59.2 ± 36.3 +	38.9 ± 24.8 #
**MVPA**	107.7 ± 92.3	114.0 ± 96.2	102.8 ± 91.2	176.5 ± 72.1 #	183.5 ± 77.4 *+	169.8 ± 70.8 #
**Weekend days**						
**Sleep**	579.4 ± 133.4	563.0 ± 153.7	594.1 ± 110.9	580.5 ± 157.5	590.2 ± 182.6	569.9 ± 120.7
**SED**	354.6 ± 197.5 *	380.2 ± 208.0	329.1 ± 191.2	509.3 ± 151.4 #*	525.0 ± 173.2 #	492.0 ± 133.4 #
**LPA**	405.5 ± 265.0 *	399.9 ± 293.8	405.7 ± 241.2	192.3 ± 98.4 #*	176.7 ± 105.7 #	206.2 ± 70.8 #
**MPA**	73.0 ± 60.9 *	70.3 ± 51.1	76.9 ± 70.8	114.9 ± 51.5 #*	97.9 ± 47.4 #+	131.8 ± 52.7 #
**VPA**	27.2 ± 35.5 *	26.3 ± 35.4	27.4 ± 34.8	44.1 ± 32.2 #*	45.3 ± 34.4 #	41.9 ± 29.9 #
**MVPA**	98.9 ± 91.5 *	93.8 ± 82.1	104.4 ± 101.0	159.1 ± 72.9 #*	143.2 ± 73.1 #	173.8 ± 72.8 #

Data are presented as mean ± SD. CF: Cystic Fibrosis, SED: sedentary time, LPA: light physical activity, MPA: moderate physical activity, VPA: vigorous physical activity, MVPA: moderate-to-vigorous physical activity. * Significant difference between week and weekend days. # Significant difference between cut-points (*p* ≤ 0.05). + Significant sex difference (*p* ≤ 0.05). Children achieved 4.5 ± 0.9 valid weekdays and 1.8 ± 0.4 valid weekend days.

**Table 3:** Change in time spent asleep and in each activity intensity using the CF-specific relative to generic cut-point.

Behaviour	Coefficient	*p*	95% Confidence Interval
Sleep	−31.4	0.019 *	[5.3–57.6]
SED	159.3	0.0001 *	[−229.3–−89.2]
LPA	−195.1	0.001 *	[111.9–278.2]
MPA	52.8	0.002 *	[−73.5–−32.3]
VPA	14.3	0.002 *	[−23.3–−5.3]
MVPA	67.1	0.0001 *	[−95.3–−39.0]

Coefficients represent change in minutes when applying CF-specific cut-points in comparison to traditional cut-points. SED: sedentary time, LPA: light physical activity, MPA: moderate physical activity, VPA: vigorous physical activity, MVPA: moderate-to-vigorous physical activity. * Significant difference (*p* ≤ 0.05).

## Data Availability

Data are not publicly available due to CGPR regulations and to protect individual privacy but are available from the corresponding author on reasonable request.
